# Exploring the Role of Phenolic Compounds in Chronic Kidney Disease: A Systematic Review

**DOI:** 10.3390/molecules29112576

**Published:** 2024-05-30

**Authors:** Filipa Baptista, Jessica Paié-Ribeiro, Mariana Almeida, Ana Novo Barros

**Affiliations:** 1 Centre for the Research and Technology of Agro-Environmental and Biological Sciences, CITAB, University of Trás-os-Montes and Alto Douro, UTAD, 5000-801 Vila Real, Portugal; 2 CECAV—Animal and Veterinary Research Centre, University of Trás-os-Montes and Alto Douro, Quinta de Prados, 5000-801 Vila Real, Portugal; jessicapaie@utad.pt (J.P.-R.); mdantas@utad.pt (M.A.)

**Keywords:** nephrotoxicity, diabetes, renal injury, herbal medicine, chronic liver diseases

## Abstract

Chronic kidney disease (CKD) presents a formidable global health concern, affecting one in six adults over 25. This review explores the potential of phenolic compounds in managing CKD and its complications. By examining the existing research, we highlight their diverse biological activities and potential to combat CKD-related issues. We analyze the nutritional benefits, bioavailability, and safety profile of these compounds. While the clinical evidence is promising, preclinical studies offer valuable insights into underlying mechanisms, optimal dosages, and potential side effects. Further research is crucial to validate the therapeutic efficacy of phenolic compounds for CKD. We advocate for continued exploration of their innovative applications in food, pharmaceuticals, and nutraceuticals. This review aims to catalyze the scientific community’s efforts to leverage phenolic compounds against CKD-related challenges.

## 1. Introduction

Chronic kidney disease (CKD) is a widely prevalent and grave issue that has a detrimental impact on human health, curtails lifespan, and escalates healthcare expenditures globally [1]. Chronic kidney disease has become a significant public health concern worldwide due to its increasing incidence and prevalence in both developed and developing nations. Notably, the kidneys show a more significant age-associated chronic pathology compared to other organs, such as the brain, liver, and heart. According to recent studies, it has been found that a substantial percentage of the adult population above the age of 25, precisely one in six individuals, is affected by CKD to some extent. Moreover, the incidence of CKD is known to increase with age, which has resulted in a staggering 1.2 million deaths and 28.0 million years of lives lost annually [2,3]. It is noteworthy that this disease is projected to become the fifth leading cause of death worldwide by 2040, manifesting as one of the largest expected increases in any major cause of death [4].

Studies have shown notable disparities in CKD prevalence among various racial and ethnic populations. For example, African Americans, Hispanic Americans, and Indigenous populations tend to have higher rates of CKD compared to Caucasians [5]. These disparities are influenced by a multitude of factors, including genetic predisposition, socioeconomic status, access to healthcare, and cultural practices [5,6].

Furthermore, CKD demonstrates distinct patterns in terms of sex-based differences. While both men and women are susceptible to CKD, there are variations in its prevalence and disease progression between the sexes. While men may exhibit a slightly higher prevalence of CKD in certain populations, women often experience faster progression to end-stage renal disease (ESRD) once diagnosed with CKD [7,8]. These sex-specific differences underscore the importance of considering gender-related factors in CKD research and management strategies.

**Figure 1:**
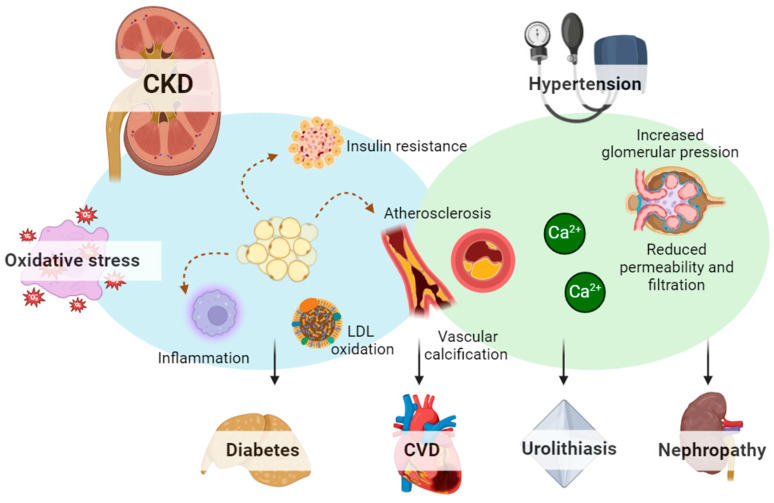
Chronic kidney disease-related conditions.

There are many causes and consequences of chronic kidney disease, including those that are common and well researched, such as diabetes, obesity, and cardiovascular disease, as well as complications due to oxidative stress, as presented in Figure 1. However, causation in chronic kidney disease is not yet fully understood. The structural features of CKD include increased interstitial fibrosis, tubular atrophy, renal vasculopathy, glomerulosclerosis, and reduced renal regenerative capability. Atherosclerosis, an arterial disease, is very prevalent in CKD patients and is the leading cause of cardiovascular disease. It is mainly characterized by low levels of high-density lipoproteins (HDLs) and increased plasma triglycerides because of very low-density lipoprotein (LDL) accumulation. Oxidative stress and inflammation aid in the development and progression of atherosclerosis and consequently of CKD. In recent years, several reports have shown indications of a rise in oxidative stress among patients undergoing renal replacement therapy and hemodialysis. However, the importance of increased oxidative stress in renal patients needs further clarification through clinical endpoint studies. This is especially crucial since studies on antioxidant treatments to prevent vascular and other diseases in non-renal patients have produced ambiguous results.

In 2015, United Nations member countries rallied around a common objective: the Sustainable Development Goals (SDGs). A pivotal health-related target within these goals is a reduction in mortality due to non-communicable diseases (NCDs) by one-third by 2030 [5]. Chronic kidney disease (CKD) has emerged as a significant global public health challenge, evidenced by its escalating incidence and prevalence rates, which have surged by approximately 5 percent over the last four years [6].

Hence, it becomes imperative to delve into the pathophysiological mechanisms underlying CKD to unearth novel therapeutic avenues capable of either preventing or decelerating the progression of this disease.

Over the past decade, there has been a remarkable increase in interest among both consumers and the scientific community regarding phenolic compounds and their antioxidant activity. This upsurge can be attributed to epidemiological studies that have established a clear link between the consumption of diets enriched with natural antioxidants and a reduced risk of diseases associated with oxidative stress, including but not limited to cancer and cardiovascular disease [9].

In the current review, we will discuss the explored effects of phenolic compounds on the treatment and progression of CKD and related diseases.

### 1.1. Phenolic Compounds

Phenolic compounds, a diverse group of plant-derived phytochemicals, encompass various classes, each with unique chemical structures and biological activities. These compounds play crucial roles in plant defense mechanisms and contribute significantly to human health due to their antioxidant, anti-inflammatory, and other bioactive properties [10,11,12].

Flavonoids, vital secondary metabolites within plants, exhibit a widespread presence across a plethora of botanical sources, including fruits, vegetables, herbs, stems, cereals, nuts, flowers, and seeds [13,14,15]. With over 10,000 distinct compounds identified to date, flavonoids showcase a remarkable diversity [16,17]. Their manifold biochemical and antioxidant properties render them potent agents in combating various ailments, such as cardiovascular disease, cancer, and neurodegenerative disorders [18,19,20,21]. Moreover, flavonoids contribute to a broad spectrum of health benefits and serve as indispensable constituents in nutraceuticals, pharmaceuticals, and medicinal and cosmetic formulations [22]. This multifaceted utility primarily stems from their anti-inflammatory, antioxidant, anticarcinogenic, and antimutagenic attributes, alongside their capacity to modulate crucial cellular enzyme functions [23,24].

Flavonoids exhibit remarkable structural diversity, leading to their classification into various types based on their chemical structure, degree of unsaturation, and carbon ring oxidation. These include flavones, flavanones, isoflavones, flavonols, chalcones, flavanols, and anthocyanins, each of which enjoys wide distribution throughout nature [25]. Phenolic acids, another prominent class, are categorized into hydroxybenzoic acids (e.g., gallic acid) and hydroxycinnamic acids (e.g., caffeic acid). Widely distributed in fruits, vegetables, whole grains, and coffee, phenolic acids possess antioxidant, antimicrobial, and anti-inflammatory properties, contributing to their potential health benefits [26,27].

Tannins, characterized by their ability to bind and precipitate proteins, are subdivided into hydrolyzable tannins (e.g., ellagitannins) and condensed tannins (proanthocyanidins). Found in foods such as grapes, nuts, and tea, tannins exhibit antioxidant, antimicrobial, and anti-inflammatory activities and are associated with various health benefits [28,29].

Lignans, abundant in flaxseeds, sesame seeds, and whole grains, are phytoestrogens known for their potential hormone-balancing effects. These compounds possess antioxidant and anti-inflammatory properties and have been studied for their potential role in reducing the risk of hormone-related cancers and cardiovascular diseases [30,31].

Stilbenes, such as resveratrol, found in grapes and red wine, are known for their cardioprotective and anti-aging effects. Resveratrol in particular has garnered significant attention for its antioxidant, anti-inflammatory, and potential anticancer properties [32,33].

### 1.2. Phenolic Compounds and Their Potential Therapeutic Benefits in Chronic Kidney Diease

Phenolic compounds have garnered attention for their potential therapeutic benefits in chronic kidney disease (CKD) [34,35]. These compounds encompass a diverse array of phytochemicals, including flavonoids, phenolic acids, tannins, and lignans, known for their antioxidant and anti-inflammatory properties [36,37].

In the context of CKD, where oxidative stress and inflammation play pivotal roles in disease progression, phenolic compounds offer promise as natural agents capable of mitigating these pathological processes [38,39,40]. Their ability to scavenge free radicals and modulate inflammatory pathways makes them attractive candidates for adjunctive CKD therapy [40,41,42].

Research suggests that phenolic compounds may exert renoprotective effects according to several mechanisms [34,43,44]. Firstly, their antioxidant properties help counteract the oxidative stress burden imposed on renal tissues in CKD, thereby preserving renal function and attenuating disease progression. Additionally, phenolic compounds possess anti-inflammatory properties, which can mitigate the inflammatory cascade implicated in CKD pathogenesis [34,42,45].

Moreover, phenolic compounds exhibit potential in ameliorating other CKD-associated complications, such as cardiovascular disease, by modulating lipid metabolism, improving endothelial function, and reducing arterial stiffness [38,41,46].

While much of the evidence supporting the beneficial effects of phenolic compounds in CKD is derived from preclinical studies and epidemiological observations, ongoing research endeavors aim to elucidate their specific mechanisms of action and therapeutic potential in human subjects [47].

In summary, phenolic compounds represent a promising avenue in the quest for novel CKD therapies, offering the potential to mitigate oxidative stress, inflammation, and associated complications. Further research is warranted to elucidate their precise mechanisms of action and establish their efficacy and safety profiles in CKD management [47,48].

## 2. Results

**Figure 2:**
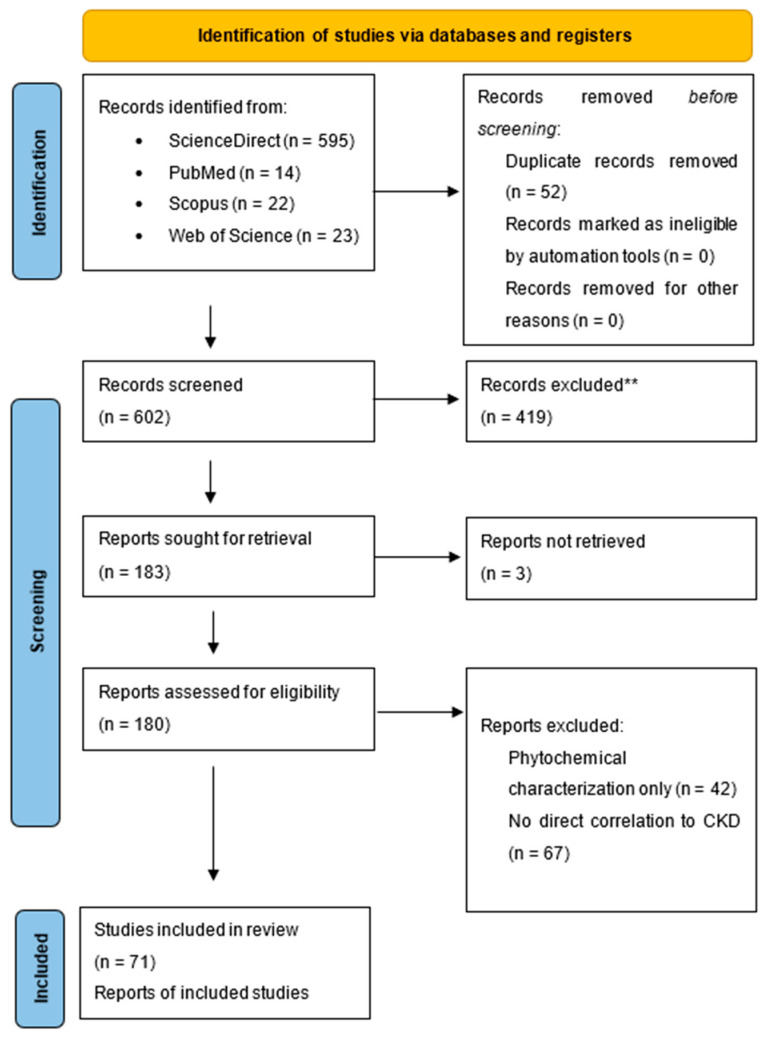
PRISMA 2020 flow diagram for new systematic reviews which included searches of databases and registers only.

A flow diagram summarizing the literature search process, screening, and the selection of potential studies is depicted in Figure 2. Initially, 654 papers were retrieved through keyword searches and Boolean operators. Following the application of exclusion criteria within the databases, the number of studies was reduced by 72%, resulting in a total of 183 scientific articles. From this pool, 71 articles were ultimately selected for further analysis. Studies briefly mentioning chronic kidney disease without clinical studies were excluded.

The analysis revealed a sharp rise in publication numbers, particularly prominent in 2020 and 2022, with Brazil emerging as the most prolific contributor. Notably, a significant portion of the literature pertaining to herbal medicine and chronic kidney disease was centred around rat models. In the Discussion section, we elucidate the primary findings extracted from our comprehensive literature review. These results are systematically categorized into thematic clusters to enhance the clarity of the current research landscape. Furthermore, we embark on an exploration of the broader implications of these discoveries, underscoring their pivotal role in advancing our understanding of physiological mechanisms and potential therapeutic avenues.

## 3. Discussion

Chronic kidney disease (CKD) is a growing health concern worldwide. While treatment options exist, managing CKD often involves a multi-pronged approach, including dietary modifications. Research hints at the potential benefits of dietary interventions in slowing the progression of CKD and improving the overall health outcomes for patients. For instance, the study by Choi et al. [49] concluded that individuals who consumed a plant-based diet, rich in polyphenols, were less likely to experience a deterioration of kidney function, especially among participants with initial-stage CKD characterized as mild. Additionally, extra virgin olive oil (EVOO), also rich in phenolics, might improve kidney function and reduce cardiovascular risks in CKD patients, as suggested by Romani et al. [50] and Marrone et al. [51]. While Silva et al. [52] found limited evidence for EVOO’s impact on specific CKD biomarkers in healthy individuals, the research on CKD patients offers promise. Jespersen et al. [53,54] determined a possible association between moderate wine consumption and a lower prevalence of CKD. Currently, Anvarifard et al. [55] is assessing the effectiveness of propolis supplementation in 44 eligible CKD patients through a multi-centred, randomized, double-blind, placebo-controlled clinical trial. If the results of this study reveal the remarkable effectiveness of propolis in improving the quality of life and clinical outcomes in patients with CKD, this compound may reach a new milestone as an adjunctive therapy for CKD, and it opens a new window for further studies.

By exploring these studies, we aim to shed light on potential dietary strategies that, alongside traditional medical management, can contribute to a more comprehensive approach to CKD management. Nonetheless, it is critical to understand CKD stands as a complex health challenge, exacerbated by various contributing factors.

### 3.1. The Role of Oxidative Stress in Chronic Kidney Disease Pathogenesis

Oxidative stress plays a pivotal role in the pathogenesis of chronic kidney disease (CKD), highlighting the imbalance between reactive oxygen species (ROS) and antioxidant defenses, which initiates a cascade of renal tissue damage [14,15]. This stress can inflict damage on DNA, exacerbating conditions such as CKD.

**Figure 3:**
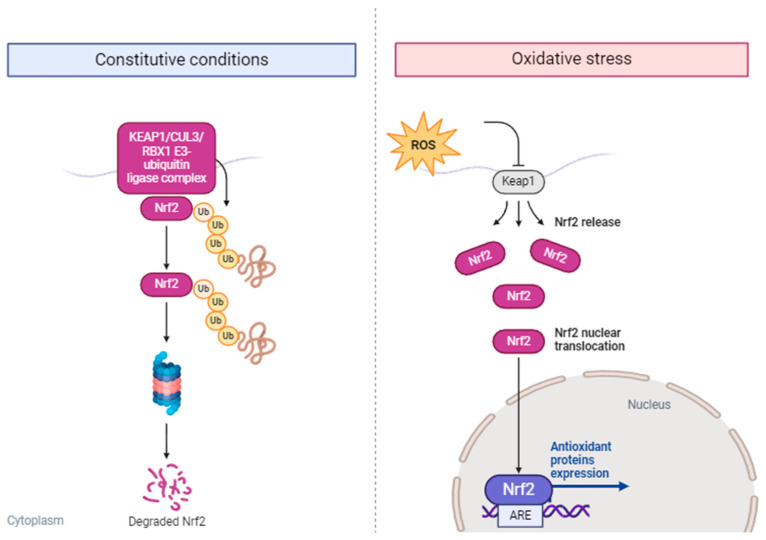
Oxidative stress: Nrf2 signaling pathway (adapted from Hammad et al. [56]).

The nuclear factor (erythroid-derived 2)-like factor 2 (Nrf2) pathway serves as a defense mechanism against oxidative stress, as illustrated in Figure 3 [56]. Under normal conditions, Nrf2 is sequestered in the cytoplasm by an inhibitory protein known as Kelch-like ECH-associated protein 1 (Keap1). However, when cells encounter oxidative stress, Keap1 releases Nrf2, allowing it to translocate to the nucleus. Once inside the nucleus, Nrf2 binds to specific DNA sequences known as antioxidant response elements (AREs), facilitating the expression of various antioxidant and detoxification enzymes [16]. These enzymes play a crucial role in neutralizing ROS and safeguarding cells from oxidative damage.

The activation of Nrf2 by oxidative stress indirectly inhibits nuclear factor kappa B (NF-κB) by upregulating the expression of antioxidant enzymes that scavenge ROS, thereby mitigating the stimuli for NF-κB activation [17]. Upon activation, NF-κB initiates the expression of genes involved in inflammation, including cytokines and adhesion molecules [18]. While these inflammatory responses can be helpful in the short term for fighting infections, chronic activation of NF-κB can contribute to tissue damage and worsen existing conditions like CKD. Additionally, factors like transforming growth factor beta (TGF-β) and tumor necrosis factor alpha (TNF-α) can also influence NF-κB activity. When TNF-α binds to its receptor, it triggers a signaling cascade that leads to NF-κB activation and promotes inflammatory responses [57]. In some cell types, TGF-β can actually suppress NF-κB activity, potentially mitigating inflammation. However, in other contexts, TGF-β can also activate NF-κB, contributing to processes like fibrosis observed in CKD [58]. Polyphenols like curcumin [57], epigallocatechin-3-gallate (EGCG) [58], resveratrol [59], a synthetic caffeamide derivative [60], and lignophenol [61] are believed to exert their beneficial effects by modulating these pathways, as seen for polyphenol-rich foods and drinks, such as dark chocolate [62,63], *Clitoria ternatea* flower petal extract [64,65], açaí [66,67], mushrooms [68,69], corn silk [70], sorghum [71], probiotic drinks [72], and unfermented grape juice [73]. They can activate Nrf2, promoting the antioxidant defense system, and/or inhibit NF-κB, TGF-β, and/or TNF-α, thereby reducing inflammation and the associated oxidative stress in CKD patients [74]. Indeed, the interactions between these pathways are intricate and remain the subject of ongoing research. Nevertheless, this simplified explanation offers a foundational understanding of how these pathways intersect and how polyphenols may potentially intervene in this interplay to mitigate the burden of oxidative stress in CKD.

### 3.2. Diabetes and Dietary Sugar Consumption

Uncontrolled diabetes leads to chronically elevated blood sugar (hyperglycemia). This hallmark feature directly damages the delicate filtration units (glomeruli) within the kidneys. Initially, the kidneys attempt to compensate for this stress by working harder, leading to an increased glomerular filtration rate (GFR). However, this hyperfiltration further strains the kidneys over time. Additionally, hyperglycemia promotes the leakage of protein (albumin) into the urine (proteinuria), an early indicator of kidney damage [75,76]. It should not be neglected how high blood sugar triggers inflammatory responses and accelerates the formation of scar tissue (fibrosis) within the kidneys, ultimately leading to impaired kidney function, while also considering the local variations in the mechanical properties [77] at specific organ regions, which can lead to different liver states in both health and disease [78]. Excessive sugar intake, particularly refined sugars and sugar-sweetened beverages, significantly contributes to hyperglycemia. This dietary habit not only increases the risk of developing type 2 diabetes but also potentiates CKD in diabetic individuals [76]. Furthermore, high sugar consumption often coincides with a diet high in saturated fats and low in fiber, further promoting metabolic syndrome, a cluster of conditions including obesity, insulin resistance, and high blood pressure, all of which further increase the risk of CKD. While all sugars can contribute to diabetes and CKD, fructose (abundant in fruits and added to processed foods) may have a more detrimental effect on kidney health [75]. This is because fructose is primarily metabolized in the liver, where it can further contribute to dyslipidemia and oxidative stress [76]. Additionally, individual susceptibility to the effects of sugar on blood sugar and kidney function can vary. Regular monitoring of blood sugar and kidney function through urine or blood tests is crucial for early detection and intervention to prevent or slow CKD progression. Certain polyphenols might improve insulin sensitivity, leading to better blood sugar control and reducing the workload on the kidneys [79,80,81]; modulate enzymes involved in carbohydrate metabolism, potentially helping to regulate blood sugar levels [82,83]; and may reduce the risk factors for type 2 diabetes by protecting against glucolipotoxicity-induced damage [84], helping prevent diabetic nephropathy [85,86,87,88,89].

By understanding the intricate link between diabetes, sugar consumption, and CKD, we can better understand the role polyphenols play in mitigating diabetic nephropathy and CKD.

### 3.3. Hypertension and Cardiovascular Disease

Hypertension and cardiovascular disease (CVD) can significantly exacerbate chronic kidney disease (CKD) through a vicious cycle. Elevated blood pressure places strain on the delicate filtration system within the kidneys, leading to gradual damage to the glomeruli (microscopic filters) and tubules, ultimately resulting in diminished kidney function and the progression of CKD [90]. Furthermore, uncontrolled hypertension constricts blood vessels throughout the body, including those that supply the kidneys. This reduced blood flow restricts the delivery of oxygen and nutrients to the kidneys, further compromising their function.

CVD often entails chronic inflammation throughout the body [91], which can harm the kidneys by promoting scarring and a decline in kidney function. Atherosclerosis, characterized by plaque build-up in the arteries due to CVD, can also impact the arteries supplying the kidneys. This narrowing of the blood vessels due to atherosclerosis mimics the effects of hypertension, exacerbating CKD. Additionally, when the heart weakens due to CVD, it struggles to effectively pump blood, leading to fluid build-up in the body, including around the kidneys, which can exacerbate kidney damage.

Taken together, hypertension and CVD hasten the decline in kidney function [91]. The impaired waste removal ability of the kidneys further elevates blood pressure, perpetuating a detrimental cycle. This accelerated progression of CKD heightens the risk of complications such as end-stage renal disease (ESRD) and necessitates dialysis [91].

Research suggests that polyphenols may offer protective effects against CVD by enhancing blood vessel function, thereby potentially improving blood pressure [92,93,94,95,96,97,98]. They may also inhibit LDL oxidation, a pivotal step in arterial plaque formation, and reduce inflammation [99,100,101]. While evidence of the role of polyphenols in ESRD is still emerging and not as robust as that for CVD and hypertension, preliminary studies on the consumption of green tea [102,103] and coffee [104] show promising outcomes.

### 3.4. Nephrotoxicity

Nephrotoxicity, characterized by a rapid decline in kidney function due to exposure to toxins or medications, can present in diverse forms. Understanding these distinct injury patterns is essential for accurate diagnosis, effective treatment, and potentially preventing further damage. Nephrotoxicity arises from disruptions in the blood flow to the kidneys, leading to a reduced glomerular filtration rate (GFR), resulting in diminished urine output and elevated blood urea nitrogen (BUN) and creatinine levels [105]. Various toxins and medications, including antibiotics, like aminoglycosides (gentamicin, tobramycin) and certain cephalosporins (cephalexin), cisplatin, and other chemotherapeutic drugs commonly used for cancer treatment, as well as environmental toxins like heavy metals (cadmium, lead, mercury) and certain industrial chemicals, can instigate this type of injury, accumulating in the kidneys and damaging the tubules and surrounding tissue [105,106]. Additionally, although less prevalent, nephrotoxicity can occur due to crystal formation within the tubules, obstructing urine flow and inducing inflammation.

Research indicates that polyphenols may offer protective effects against nephrotoxicity. For instance, elderflower [107] and fish oil [108] significantly enhanced renal activity in gentamicin-induced nephrotoxicity in rat models, while bee propolis [109,110], fig leaves [111,112], avocado, walnuts [113,114], pitaya juice [115], black soybeans [116], and other medicinal plants [113,117,118,119] may reverse the renal damage caused by anticancer agents such as adriamycin, 5-fluorouracil, and cisplatin. Furthermore, guava leaves [120], stevia residue [121,122], and ginger [123] have demonstrated efficacy in attenuating medication-induced renal atrophy.

Further research has shown that polyphenol-rich foods, including Indian plums [124], radishes [125,126], green algae [127], acacia gum [128], basil leaves [129,130], essential oils [131], coffee and olive oils [132], and red palm oil [133,134], can ameliorate kidney damage induced by various toxins. Moreover, phenolic compounds have shown promising results in preventing further heavy-metal-induced kidney injury [135,136,137]. While research suggests that some polyphenols might hold promise in safeguarding the kidneys from nephrotoxic damage, further studies are warranted to confirm their efficacy and safety in human subjects.

### 3.5. Other Related Conditions

Beyond the previously discussed conditions, CKD can also be associated with hyperuremia (high blood uric acid) and urolithiasis (kidney stones). Hyperuricemia is common in chronic kidney disease (CKD) and may be present in 50% of patients presenting for dialysis, and while it is a risk factor for CKD, it is not entirely clear whether lowering uric acid levels can prevent or slow down CKD progression in all cases. Nonetheless, research shows the polyphenols present in both moringa leaves [138] and peony flowers [139,140] can effectively reduce the serum uric acid levels in rats by regulating serum xanthine oxidase activity and renal urate transporters. In addition, polyphenol-rich foods have shown diuretic properties that could help mitigate urolithiasis [141,142,143,144,145].

### 3.6. Polyphenol-Rich Foods and Their Reported Activity

Phenolic compounds are a diverse group of secondary metabolites found in plants, characterized by their aromatic ring structures with one or more hydroxyl groups attached. The structural diversity of phenolic compounds contributes to their wide range of bioactivities, which are determined by specific structural features [47,146].

For example, the number and position of hydroxyl groups on the aromatic ring influence the antioxidant activity of phenolic compounds. Compounds with more hydroxyl groups tend to exhibit higher antioxidant potential due to their ability to donate hydrogen atoms and scavenge free radicals, thus protecting cells from oxidative damage [147,148]. This structural configuration facilitates hydrogen atom donation, effectively neutralizing free radicals and protecting cells from oxidative damage. The Free Oxygen Radical Test (FORT) and total antioxidant capacity (TAC) assays serve as valuable tools for quantifying this enhanced antioxidant capacity [149,150].

Similarly, the presence of conjugated double bonds in the aromatic ring system enhances the anti-inflammatory properties of phenolic compounds. This structural feature allows phenolic compounds to modulate inflammatory pathways by inhibiting the production of pro-inflammatory mediators and cytokines like interleukin-6 (IL-6) [47,151]. By mitigating the activity of these inflammatory signaling molecules, phenolics can demonstrably decrease the levels of C-reactive protein (CRP) and the erythrocyte sedimentation rate (ESR)—established biomarkers of systemic inflammation [152]. Superoxide dismutase (SOD), an enzymatic defense mechanism against free radicals, may be indirectly influenced by certain phenolic structures. However, their primary impact is likely exerted on malondialdehyde (MDA), a byproduct of lipid peroxidation—a process triggered by free radicals. By effectively scavenging free radicals, phenolics can significantly reduce MDA levels, signifying decreased oxidative damage [153].

Additionally, the presence of certain functional groups, such as methoxy or glycoside groups, can affect the solubility and bioavailability of phenolic compounds, thereby influencing their biological activity. These structural modifications may also impact the interactions of phenolic compounds with enzymes, receptors, and other cellular targets, further contributing to their diverse bioactivities [154,155].

Overall, the structure of phenolic compounds plays a crucial role in determining their bioactivity, making them promising candidates for various applications in medicine, nutrition, and agriculture. Understanding the structure–function relationships of phenolic compounds can help in the design and development of novel therapeutic agents with enhanced efficacy and safety profiles [156,157].

Found abundantly in fruits, vegetables, grains, and beverages like tea and wine, these compounds are known for their antioxidant and anti-inflammatory properties. Their presence in foods underscores the importance of a varied and colorful diet for overall health and well-being. An overview of the phenolic compounds and their reported bioactivity in the studies included in this systematic review is presented in Table 1.

### 3.7. Notes on the Excluded Research

This review aimed to comprehensively evaluate the current research on the use of polyphenols and polyphenol-rich foods to treat CKD and CKD-related conditions. A rigorous selection process was employed to ensure the included studies met specific criteria related to the real impact on CKD. While a total of 183 studies were identified through our search strategy, 112 studies were ultimately excluded from this review.

This section details the rationale behind the exclusion of these studies. Understanding the limitations of the included research is crucial for interpreting the findings and identifying potential areas for future investigation. The excluded studies may have been focused on conditions or assessments not directly associated with CKD, with methodological limitations impacting the relevance of the results to this review, or may have lacked a control group.

The review focused on exploring the potential benefits of phenolic compounds in chronic kidney disease (CKD) and related conditions. Consequently, studies investigating the adverse health effects of specific phenolic compounds, such as *p*-Cresol, were deliberately excluded. *p*-Cresol, a methylated phenolic compound and gut microbiome metabolite, has been associated with documented negative effects on kidney function. While phenolic compounds encompass a diverse group with several health benefits, certain compounds like *p*-Cresol can have detrimental effects. It is crucial to note that *p*-Cresol is not typically derived from plants. Although many phenolic compounds are sourced from plant-based origins, *p*-Cresol primarily originates from two main sources. Firstly, it is produced through industrial processes, conventionally extracted from coal tar, a by-product of coal roasting for coke production. Secondly, *p*-Cresol is generated within the gut microbiome, through the bacterial fermentation of proteins in the large intestine. Given these origins and its established negative health impacts on the kidneys, *p*-Cresol was deemed irrelevant for the purposes of exploring the benefits of plant-based phenolics for CKD.

By presenting these considerations, our aim is to provide a transparent overview of the existing evidence and to identify areas where further research is needed to better understand the potential benefits of plant-based phenolics in CKD management.

## 4. Materials and Methods

This study employed a systematic selection of literature within the field of management research. The study’s overarching aims were exploratory and descriptive in nature, facilitating a comprehensive evaluation of the data and the acquisition of interpretative insights. The technical procedures employed in this research closely adhered to the systematic literature review framework, following the well-established steps of PRISMA. These steps encompassed formulating the research question (I), identifying pertinent databases (II), devising search strategies and selecting and accessing pertinent literature (III), evaluating the quality of the studies included (IV), and ultimately conducting an in-depth analysis, synthesis, and dissemination of the research findings (V).

### 4.1. Formulatation of the Research Question (I)

Research Question: What is the scientific research scenario on the use of phenolic compounds in the prevention, treatment, and management of chronic kidney disease?

### 4.2. Searched Databases and the Search Strategies (II)

Conducting a systematic review depends on the scope and quality of the included studies. Thus, to recover scientific articles of proven quality, we sought to use only electronic bases that retrieve journals that perform peer reviews of a manuscript, which were national and international. The bases used were Web of Science, Scopus, PubMed, and ScienceDirect. Next, each database’s descriptors and the Booleans used as search guidance were determined. The search method varied several times among databases because they have some peculiarities. Boolean operators were used to establish the selection criteria and thus retrieve the maximum number of papers related to the theme. All bases were last consulted on the 11th of February. The descriptors used as search guidance in each database were determined. Keywords were established according to the selection criteria, and thus the maximum number of papers related to the theme was retrieved. When searching, the AND operator was inserted between two words to obtain articles that contain both (e.g., “phenolic compounds” AND “chronic kidney disease”). Keywords between quotation marks were used to refine the search. Therefore, articles were retrieved only if the words appeared together. In all the databases, no time/date limit was imposed. After obtaining the results, only scientific articles were selected, excluding books and book chapters, review articles, conference papers, notes, letters, errata, and editorials.

The searches in the Scopus, PubMed, Web of Science, and ScienceDirect databases were similar. The following keywords and combinations were used through Booleans to refine the search and exclude irrelevant articles. The search was not restricted (All fields) in the Web of Science database and restricted to the title, abstract, and keywords in all the other databases to the following combination: “phenolic compounds” AND “chronic kidney disease”. After obtaining the results, only scientific articles were selected, excluding books and book chapters, review articles, notes, letters, conference papers, errata, and editorials. Duplicates in the same database were discarded.

### 4.3. Inclusion and Exclusion Criteria (III)

The articles retrieved from the databases were selected according to the following criteria:

Only studies where phenolic compounds were used to prevent, treat, or manage CDK and CKD-related conditions were selected. After being selected according to the mentioned criteria, the articles were grouped into different knowledge fields: Oxidative stress and signaling pathways, diabetes and dietary sugar consumption, hypertension and cardiovascular disease, nephrotoxicity, and other related conditions. Records were marked as ineligible when they only briefly mentioned CKD and made no significant remarks on how the study impacted this disease, namely studies that only studied the phytochemical profile of food matrices.

### 4.4. Critical Analysis of the Selected Studies (IV)

The articles chosen in the previous step were read in full to extract relevant aspects of the objectives, methodology, results, and conclusions. This analysis also considered the quality of the studies, which required a detailed description of the methodology and conclusive results with a thorough discussion.

### 4.5. Summary of the Results (V)

The presentation of the results focused on describing the main characteristics of the studies, highlighting the effects of phenolic compounds on CKD, either in preventing, treating, or managing the disease.

## 5. Conclusions

Chronic kidney disease (CKD) is a global health issue affecting around 850 million individuals worldwide, particularly in low- and middle-income nations. Dietary polyphenols have emerged as a promising avenue for the treatment and management of CKD. Recent studies have shed light on the mechanisms through which polyphenols can alleviate CKD-related conditions. However, despite valuable insights from the existing research, several unanswered questions and knowledge gaps persist.

Exploring potential avenues for future research is essential to advance our understanding of how CKD affects different organs. This includes investigating the long-term effects of dietary interventions, delving into the underlying mechanisms of the observed benefits, and conducting larger and more diverse human clinical trials. Addressing these research gaps is pivotal in developing more effective strategies for managing and potentially preventing CKD.

Polyphenols and other food phenolics have garnered increasing interest in the scientific community due to their potential to promote health and well-being. However, more research is needed to understand the metabolism and availability of these compounds in the body, essential factors for unlocking their beneficial effects on human and animal health. Future research should focus on exploring new cultivars to identify novel compounds and elucidate their mechanisms for medical and pharmaceutical purposes. Phytochemical characterization is indispensable to driving progress in discovering and developing new drugs based on natural compounds.

Recent research suggests that polyphenols may hold significant promise in CKD care, offering potential advantages such as slowing disease progression, enhancing kidney function, and mitigating the cardiovascular risks associated with CKD. However, the successful integration of polyphenols into CKD treatment necessitates patient education and active participation to ensure adherence to dietary changes.

This review highlights the importance of a collaborative, multidisciplinary approach involving nephrologists, dietitians, and other healthcare professionals to delivering comprehensive CKD care. Synthesizing diverse research findings not only advances our understanding of CKD but also serves as a practical guide for clinicians and researchers navigating the complexities of CKD treatment and management.

Ultimately, this synthesis contributes to the ongoing discourse on refining strategies to enhance the quality of life for CKD patients worldwide, underscoring the promising role of dietary polyphenols in this endeavor. Therefore, in-depth research into exploiting new cultivars, understanding the mechanisms of action, and enhancing accessibility to CKD treatments is imperative for fostering a more sustainable future and promoting global health and well-being.

## Figures and Tables

**Table 1:** Phenolic compounds and their reported bioactivity in the reviewed studies.

Compound		Reported Bioactivity	
Hydroxytyrosol	[50]	↑ e-GFR after 9 weeks ↓ oxidative stress ↓ uric acid levels	[50]
Oleacin (10-hydroxy-oleocanthal) Hydroxytyrosol Tyrosol Elenolic acid (and derivatives)	[51]	↓ serum levels of creatinine, azotemia, albuminuria, uric acid ↓ FORT level, ESR, and CRP, TNF-α, and IL-6 serum levels	[51]
Hydroxytyrosol (and derivatives)	[52]	Improvement in the urinary proteomic biomarker of disease over a relatively short period in a healthy population	[52]
Hydroxybenzoic and hydroxycinnamic acids Stilbenes	[54]	↓ hypertension, HDLs	[53]
Curcumin	[57]	↓ NF-kB mRNA expression and hsCRP plasma levels	[57]
Epigallocatechin-3-gallate	[58]	Prevention of EMT in renal tubular cells induced by the crosstalk between TGF-β1 and β-catenin signaling	[58]
Resveratrol	[59]	There was no effect of resveratrol supplementation on Nfr2 and NF-kB expression at the dosage tested	[59]
Caffeamide (derivative)	[60]	↓ oxidative stress ↓ AngII and TGF-β1 production and Smad3 phosphorylation	[60]
Lignophenol	[61]	↓ plasma BUN levels ↓ TNF-α, Ccl2, and TGF-β	[61]
Flavan-3-ols	[63]	↓ plasma levels of TNF-a	[62]
Kaempferol Caffeoylmalic acid	[65]	Prevented a decrease in GSH concentration in erythrocytes Inhibited the formation of the echinocytic form	[64]
Vanillic acid, syringic acid, p-hydroxybenzoic acid, protocatechuic acid, ferulic acid, and (+)-catechin	[67]	Prevented higher expression of MCP-1 and ICAM-1 ↓ TNF-*α* concentration in cells ↓ protein carbonylation	[66]
1-octene-3-alcohl and butylated hydroxytoluene	[69]	↓ BUN and Scr ↓ content of ROS in renal tissues ↓ oxidative stress ↑ SOD and GSH-PX	[68]
Gallic acid, catechin and derivatives, and rutin and derivatives	[70]	↓ blood urea nitrogen and serum creatinine	[70]
Luteolinidin and 5- methoxyluteolinidine	[71]	↓ oxidative stress ↑ serum SOD and TAC ↓ serum MDA decreased	[71]
Hydroxycinnamic acids and flavonoids and caffeic acid derivatives	[110]	↑ SOD 1 activity ↓ IL-1β, TNF-α, and IL-6 production	[72]
Hydroxybenzoic and hydroxycinnamic acids, stilbenes	[54]	↓ LDLs and cholesterol ↓ oxidative DNA damage	[73]
Curcumin	[79]	↓ serum urea and creatinine ↓ HO1 and INOS mRNA expression ↑ GSH concentration, GR, CAT, and SOD activities ↓ LPO and DNA fragmentation	[79]
Ellagitannin geraniin	[80]	↓ relative weights of pancreas, liver, heart, and aorta ↓ plasma glucose ↓ TG, non-HDLs and total cholesterols, ALT, AST, CK, and Cr	[80]
Protocatechuic acid	[81]	↓ BMI ↓ SOD activity in liver and muscle homogenates ↑ GSH activity in muscle ↓ MCP-1, IL-1b, CRP	[81]
3,4-dihydroxybenzoic acid	[82]	↓ TNF-α expression by 0.12 times	[82]
Ferulic and benzoic acid derivatives, quercetin derivatives, flavonone glycosides	[83]	↓ cholesterol, LDLs, and plasma TGCs	[83]
7-O-Galloyl-d-Sedoheptulose	[84]	↓ serum levels of triglycerides, total cholesterol, LDL/VLDL-cholesterol, NEFAs, and TBA-reactive substances ↓ serum levels of ALT, AST, creatinine, and urea nitrogen	[84]
Thymol	[85]	↓ urinary glucose, urinary urea, and urinary protein ↓ TBARS and LOOH ↑ SOD, catalase, GPx, GST, and GR ↓ SREBP-1c, TGF-b, and VEGF proteins	[85]
Protocatechuic acid	[89]	↓ insulin Improved AGE expression and histological changes in both glomerular hypertrophy and interstitial crushing	[86]
Protocatechuic acid, gallic acid, catechin, gallocatechin, and rutin	[87]	↓ triacylglycerol and total cholesterol levels ↓ BUN, blood creatinine, and blood pressure ↓ ratio of urine albumin/urine creatinine Ameliorated mesangial fibrosis in part via the Ras/PI3K/Akt signaling pathway.	[87]
Epicatechin	[88]	↓ BUN, total cholesterol, and triglycerides ↓ MDA ↑ SOD, GSH, and catalyze Regeneration of tubular epithelium, inhibition of necrosis and hemorrhages, and recovery of atrophic glomeruli	[88]
Gallic acid, catechin and derivatives, and rutin and derivatives	[70]	↓ systolic and mean arterial BP ↑ plasma and urinary nitrite and nitrate	[92]
Gallic acid, catechin and derivatives, and rutin and derivatives	[70]	Recovery in the kidney morphology	[93]
Catechin	[96]	↑ GSH and the GSH2/GSSG ratio	[94]
Stigmasterol, betulinic acid	[97]	↓ lipid peroxidation Antihemolytic effect ↓ blood pressure ↓ kidney hypertrophy ↑ CAT and SOD in the kidney	[95]
Hyperoside, quercitrin, caftaric acid, gentisic acid, caffeic acid and chlorogenic acid	[99]	↓ blood pressure ↓ blood levels of uric acid, urea, creatinine, and urine levels of NAG ↓ TNF-α	[99]
Gallic acid, catechin, epicatechin, rutin, quercetin, and kaempferol	[100]	↓ plasma concentrations of ALT, AST, and ALP ↑ SOD ↓ MDA, NO, and APOP ↑ catalase and SOD	[100]
Caffeic acid, chlorogenic acid, anthocyanins, p-coumaric acid, ferulic acid, o-coumaric acid, quercetin, gallic acid, rutin, catechin	[98]	↓ CRP The activity of SOD and catalase enzymes, as well as the concentration of MDA, did not differ from the placebo group	[101]
Epigallocatechin, catechin, and epicatechin gallate	[102]	↓ fibrinogen levels, protein expression of p22phox, and hsCPR	[102]
Epigallocatechin, catechin, and epicatechin gallate	[102]	↓ pERK1/2 phosphorylation ↓ oxLDL plasma levels	[103]
Catechin, epicatechin, quercetin, caffeic acid, and ferulic acid	[107]	↓ urinary NAG activity ↓ renal histopathological lesion ↓ urea and serum creatinine	[107]
Ferulic acid		↓ serum BUN and creatinine levels ↓ urinary albumin concentration and urinary NAG activity ↑ CAT activity ↑ RvE1 concentration ↑ PPAR-γ gene expression	[108]
Ferulic acid	[110]	Prevented disruption of the normal renal architecture ↓ creatinine, BUN, LDH, TNFa, and KIM-1 levels	[109]
Gallic acid, chlorogenic acid, syringic acid, (+)-catechin, (β)-epicatechin, and rutin	[112]	↓ MDA, Hyp, and Hcy	[111]
P-couramic, caffeic, and ferulic acids	[114]	↓ serum MDA, lipid peroxidation ↑ plasma catalase and total antioxidant capacity ↑ Cr ↓ chromosomal aberrations in bone marrow cells and sperm shape abnormalities	[113]
Betacyanins	[115]	↓ plasma creatinine, BUN, and NGAL ↓ tubular damage ↑ MDA levels ↓ Nrf2 levels ↑ NO_2_^−^/NO_3_^−^ levels	[115]
Isoflavones (daidzein, daidzin, genistin, biochanin A, and glycitein)	[116]	↓ cytoplasmic IFN- γ expression ↓ Casp-3 expression ↓ IL-6, IL-1b, TGF-b1, TLR-4, F4/80 and TNF-a in kidneys ↓ BUN and UA ↑ CAT	[116]
Flavonoids and terpenoids	[117]	↓ blood urea and creatinine ↑ glutathione peroxidase activity	[117]
Myricetin	[118]	↓ BUN, β2-MG, and Cys C ↑ GR activity, TAS and GPx, MDA ↓ TNF-α and IL-1β	[118]
Tannins, phenolics, flavonoids, steroid glycosides, terpenoids, and saponins	[119]	↓ serum concentrations of BUN and creatinine ↓ renal tubular and glomerular alterations	[119]
Gallic acid, (+)-catechin, (–)-epicatechin, and ellagic acid	[120]	↓ ALT and AST activities ↓ MDA, APOP and NO in plasma, heart, and kidney ↓ uric acid level, and plasma creatinine level	[120]
Chlorogenic acid, diosmin, and caffeic acid	[122]	↓ urinary NGAL, endothelin-1, and clusterin levels ↓ BUN, creatinine, 8-OHda, isoprostane, adiponectin, and cystatin ↓ serum XOD, liver XOD, serum ADA, liver ADA, and serum UA levels	[121]
6–Shogaol, 6–Paradol, and 6–Gingerol	[123]	↓ plasma and blood creatinine and urea ↓ GSH level ↑ SOD level	[123]
Ferulic acid, caffeic acid and vanillic acid	[124]	↓ MDA, APOP, uric acid and creatinine levels in the blood, IL-1, IL-6, TNF-α, TGF-β1, and NF-κB ↑ catalase and SOD activities in the kidneys	[124]
Chlorogenic, vanillic, caffeic, syringic, p-coumaric, and ferulic acids	[126]	↓ serum urea, uric acid and creatinine levels ↑ kidney SOD, CAT, GPx, GST, and GR activities	[125]
Lutein and chlorophyll	[127]	↑ CAT and SOD ↓ urea, creatinine, uric acid, GGT, CK, BUN, and β2-microglobulin ↓ levels of TNFα, IL-6, IL-β, and TGF-α	[127]
Benzoic acids	[128]	↓ levels of Nrf2 and SOD1 in renal tissue Normal architecture of renal tissue (renal corpuscle, proximal and distal convoluted tubules, and collecting ducts)	[128]
Rosmarinic acid	[130]	↓ serum creatinine, BUN, and uric acid	[129]
Eugenol	[131]	↓ oxidative stress, inflammation, and apoptosis ↓ proinflammatory markers (IL6 and TNF-α)	[131]
Chlorogenic acid, hydroxytyrosol, and tyrosol	[132]	↓ creatinine, uric acids, and BUN	[132]
Pyrogallol, 4-hydroxybenzoic acid, gallic acid, and ferulic acid	[134]	↑ GSH, SOD, CAT ↓ MDA	[133]
Secoisolariciresinol diglucoside	[135]	↓ levels of NO and MPO ↑ superoxide dismutase, catalase, glutathione peroxidase, and glutathione reductase	[135]
Proanthocyanidins		↑ SOD activity ↓ MDA content, CR and BUN levels	[136]
Kaempferol and quercetin derivatives		↓ serum UA level, liver MDA, serum Cr, and serum TG	[138]
Kaempferol, luteolin, and apigenin	[140]	↓ serum UA, XOD, MCP-1, TNF-α, Cr, and BUN	[139]
Pyrrolidide amides, chalcones, and flavonols	[141]	↑ urine output ↓ calcium oxalate crystallization	[141]
Retinol and caffeoylquinic acid	[142]	Ameliorated abnormal urinary levels of calcium, oxalate, phosphate, magnesium, citrate, protein, and uric acid ↓ serum BUN, creatinine, and uric acid levels	[142]
Narirutin, neohesperidin, hesperidin, rutin and citric acid	[143]	↓ nucleation and growth and aggregation of calcium oxalate crystals ↓ renal tubular dilation and renal tissue deterioration	[143]
Flavonoids, steroidal saponin, and organic acids	[144]	Urinary excretion of total protein, urea, creatinine, sodium, potassium, calcium, and chloride	[144]
Gallic acid	[145]	↑ ACE inhibitory activity	[145]

Increased (↑), decresead (↓), C-reactive protein (CRP), erythrocyte sedimentation rate (ESR), pro-inflammatory cytokines (interleukin 6 (IL-6)), superoxide dismutase (SOD), malondialdehyde (MDA), total antioxidant capacity (TAC), tumor necrosis factor α (TNF-α), interleukin 1b (IL-1b), transforming growth factor b1 (TGF-b1), toll like receptor-4 (TLR-4), catalase (CAT), glutathione-S-transferase (GST), glutathione peroxidase (GPx), glutathione reductase (GR), renal reduced glutathione (GSH).

## Data Availability

Not applicable.
